# The crystal structures of the enzyme hydroxymethylbilane synthase, also known as porphobilinogen deaminase

**DOI:** 10.1107/S2053230X2100964X

**Published:** 2021-10-19

**Authors:** John R. Helliwell

**Affiliations:** a Department of Chemistry, University of Manchester, Manchester M13 9PL, United Kingdom

**Keywords:** hydroxymethylbilane synthase, porphobilinogen deaminase, enzyme–substrate intermediates, reaction mechanisms, structure and function

## Abstract

The enzyme hydroxymethylbilane synthase (HMBS; EC 4.3.1.8), also known as porphobilinogen deaminase, catalyses the stepwise addition of four molecules of porphobilinogen to form the linear tetrapyrrole 1-hydroxymethylbilane. Thirty years of crystal structures are surveyed in this topical review. These crystal structures aim at the elucidation of the structural basis of the complex reaction mechanism involving the formation of tetrapyrrole from individual porpho­bilino­gen units. The consistency between the various structures is assessed. This includes an evaluation of the precision of each molecular model and what was not modelled. A survey is also made of the crystallization conditions used in the context of the operational pH of the enzyme. The combination of 3D structural techniques, seeking accuracy, has also been a feature of this research effort. Thus, SAXS, NMR and computational molecular dynamics have also been applied. The general framework is also a considerable chemistry research effort to understand the function of the enzyme and its medical pathologies in acute intermittent porphyria (AIP). Mutational studies and their impact on the catalytic reaction provide insight into the basis of AIP and are also invaluable for guiding the understanding of the crystal structure results. Future directions for research on HMBS are described, including the need to determine the protonation states of key amino-acid residues identified as being catalytically important. The question remains – what is the molecular engine for this complex reaction? Thermal fluctuations are the only suggestion thus far.

## Abstract 2

Thirty years of crystal structures of the enzyme hydroxymethylbilane synthase are surveyed in this topical review. These crystal structures aim at the elucidation of the structural basis of the complex reaction mechanism involving the formation of tetrapyrrole from individual porphobilinogen units.

## 1. Introduction   

**Figure 1:**
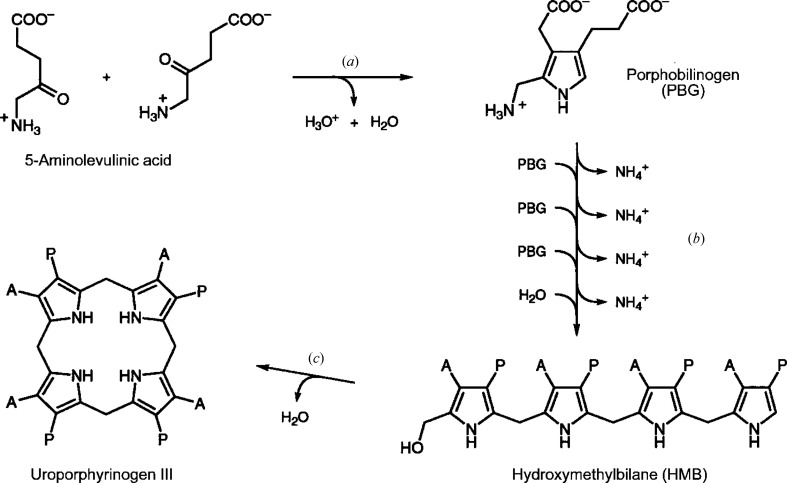
The biosynthesis of uroporphyrinogen III from 5-aminolevulinic acid. The enzymes involved are (*a*) 5-aminolaevulinic acid dehydratase, (*b*) hydroxymethylbilane synthase (HMBS) and (*c*) uroporphyrinogen III synthase. A = CH_2_COO^−^; P = CH_2_CH_2_COO^−^. Reproduced from Hädener *et al.* (1999 ▸).

**Figure 2:**
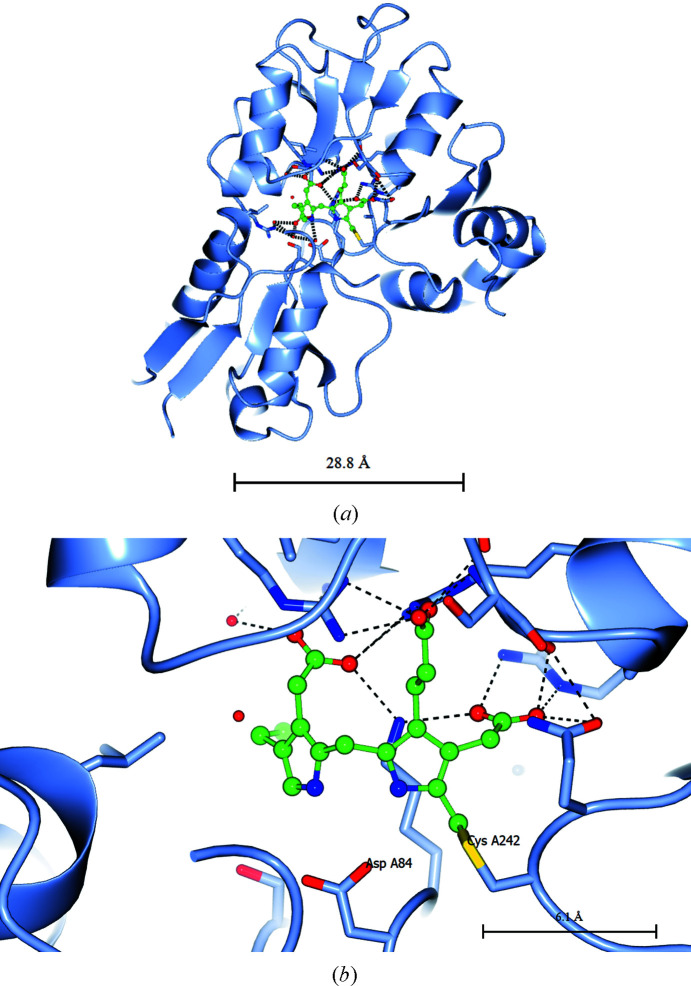
(*a*) The crystal structure of the active form of the *E. coli* HMBS enzyme in ribbon format (PDB entry 1ah5); the cofactor is in the middle of the picture. (*b*) An enlargement of the dipyrromethane cofactor; the right-hand cofactor ring (referred to in the text as C1) is covalently attached to the labelled Cys242 (Cys261 in the human enzyme). The Asp side chain (Asp84 in *E. coli* HMBS and Asp99 in human HMBS) is visible just below the cofactor towards its left-hand side. This figure was produced by *CCP*4*mg* (McNicholas *et al.*, 2011 ▸).

The course of the reaction catalysed by hydroxymethylbilane synthase (HMBS) is depicted in Fig. 1 ▸ as well as its overall position in the pathway to uroporphyrinogen III (Hädener *et al.*, 1999 ▸). Note the ammonia molecules released in each step. The crystal structure with PDB code 1ah5 (Hädener *et al.*, 1999 ▸) is depicted in Fig. 2 ▸.

## 2. Commentary on the role of the PDB data files in structural and functional studies of HMBS   

The details of each deposition are provided in Table 1 ▸. A synopsis of the role of each crystal structure is now provided. The HMBS structures studied are from multiple organisms; these are listed in Table 1 ▸. It is made clear to which organism the amino-acid numbering of the highlighted residues below refers.

*PDB entry 1pda*. This is the opening, pioneering, crystal structure. It was of the inactive form of the enzyme. Louie *et al.* (1992 ▸) suggested that the enzyme mechanism has two options: the sliding of the catalytic residue Asp84 (*Escherichia coli* numbering) or the movement of the cofactor past Asp84, in which the relative motion of the individual domains is also likely to be involved.
*PDB entry 1ah5*. This is the active form of the enzyme and was solved by selenomethionine MAD phasing using Station 9.5 at the SRS in the UK (Hädener *et al.*, 1993 ▸, 1999 ▸). This study also gave early experience of the ESRF BM14 beamline and the ESRF image-intensifier detector (Cassetta *et al.*, 1999 ▸). In Hädener *et al.* (1999 ▸), the SRS and ESRF MAD data and results are also compared.
*PDB entry 1ypn*. This was the first experimental demonstration of the active-site location. It corresponds to the 2 h time point after reaction initiation by diffusion of porpho­bilinogen (PBG) into the crystal using a flow cell monitored by time-resolved diffraction on ID09 at ESRF. The experiment was based on the K59Q HMBS mutant, experiments using which had shown a buildup of the enzyme–substrate intermediate ES_2_ (Niemann *et al.*, 1994 ▸). Extended electron density was established after 2 h in a geometric series of measured time-point data sets from 1 min to 12 h. PBG fits the 2 h extended electron density (2*F*_o_ − *F*_c_); this is likely to be the first reaction site (ES_1_) as it is proximal to Asp84. The other end of the elongated density extended towards the side chain of Arg149, which is also an important residue. The maximum peak height was around 3.8σ and the length of the peak was around 8 Å. The enzyme–substrate intermediate complexes require a covalent bond between the C2 ring of the cofactor and ring A of the polypyrrolic chain. However, the 2 h elongated density peak is not connected to the C2 ring of the cofactor; this point is discussed further below.
*PDB entry 2ypn*. This was the time-zero data set that accompanied PDB entry 1ypn from the experiment on ID09 at ESRF.
*PDB entry 1gtk*. This crystal structure determination was from diffraction data that were measured at 100 K and was compared with PDB entry 1ah5, the room-temperature crystal structure. However, the mobile loop consisting of residues 45–57, which sits close to the active site of HMBS, was not stabilized in PDB entry 1gtk. This loop was defined structur­ally in PDB entry 4htg, the *Arabidopsis* enzyme (Roberts *et al.*, 2013 ▸), as well as being offered as a predicted structure by AlphaFold DB (Jumper *et al.*, 2021 ▸).
*PDB entries 3ecr, 3eq1, 4htg, 4mlq and 4mlv* are crystal structures of HMBS from other species and organisms. PDB entries 3ecr and 3eq1 are of human HMBS and are especially interesting because the mutations responsible for the genetic-based disease acute intermittent porphyria could be directly visualized in these human HMBS crystal structures. *PDB entry 5h6o* is listed as ‘to be published’ and the PDB deposition is entitled *Porphobilinogen deaminase from Vibrio cholerae*.
*PDB entries 5ov4, 5ov5 and 5ov6* are crystal structures that each involve a mutant of the catalytic aspartic acid (Asp82 in *Bacillus megaterium* numbering). Guo *et al.* (2017 ▸) reported that ‘the only mutant, D82E, which has the whole cofactor bound in a well ordered manner is catalytically active, while the other two (D82A and D82N) are not’.

Since 2018, several groups have reported studies of ES_2_ intermediate structures. These structures are as follows.

*PDB entry 5m6r* (Pluta *et al.*, 2018 ▸) is a structure entitled *Human porphobilinogen deaminase in complex with reaction intermediate ES_2_*. Note the 3.5 Å change in the *c* unit-cell parameter compared with PDB entry 5m7f, the holo enzyme.
*PDB entry 7aak* (Bustad *et al.*, 2021 ▸) is a structure entitled *Human porphobilinogen deaminase R173W mutant crystallized in the ES_2_ intermediate state*. The maximum differences in unit-cell parameters compared with PDB entry 7aaj, the holo enzyme, are the values of *b* and *c*, which differ by 1.5 Å, *i.e.* the ES_2_ intermediate was captured with only a small unit-cell change.
*PDB entry 7cdo* (Sato *et al.*, 2021 ▸) is a structure entitled *Crystal structure of the 2-iodoporphobilinogen-bound ES_2_ intermediate form of human hydroxymethylbilane synthase*. The crystals used by Sato and coworkers are shown in their Supplementary Fig. S3. 2-Iodoporphobilinogen (2-I-PBG) is described by Sato *et al.* (2021 ▸) as a noncompetitive inhibitor. From the same group of studies is PDB entry 7ccy entitled *Crystal structure of the 2-iodoporphobilinogen-bound holo form of human hydroxymethylbilane synthase*. Note the 7.4 Å difference in the *a* unit-cell parameter compared with PDB entry 7cd0. PDB entry 7ccz is entitled *Crystal structure of the ES_2_ intermediate form of human hydroxymethylbilane synthase* and PDB entry 7ccx is entitled *Crystal structure of the holo form of human hydroxymethylbilane synthase*. Note that the *a* unit-cell parameter of PDB entry 7ccx differs by 4 Å compared with that of PDB entry 7ccy and by 11 Å compared with those of PDB entries 7ccz and 7cdo. In effect there are two unit-cell clusters here, albeit broadly similar. The use of 2-I-PBG by Sato *et al.* (2021 ▸) is interesting. They showed that it inhibited the HMBS reaction in a noncompetitive manner. Sato and coworkers reported that ‘this contrasted with reported competitive and mixed-type inhibitors, such as 2-bromo-PBG and 6-methyl-PBG, respectively, which form covalent bonds with the cofactor and oligopyrrole chain, while 2-I-PBG does not form such’. They also reported that ‘The overall structure of the 2-I-PBG-bound holo-HMBS was found to be similar to that of the inhibitor-free holo-HMBS’. Table 2 of Sato *et al.* (2021 ▸) provides comprehensive details of the interactions between pyrroles and the protein moiety in HMBS (in their four crystal structures). Fig. 8 of Sato *et al.* (2021 ▸) shows a predicted ES_1_ based on their crystal structure of human HMBS complexed with 2-I-PBG. As a historical note, bromoporphobilinogen was important in the study of the enzyme, as experiments by Warren & Jordan (1988 ▸) using bromoporphobilinogen provided conclusive evidence for the direct covalent interaction of the substrate with the dipyrromethane (DPM) cofactor.

In mid-2021 the AlphaFold Protein Structure Database (AlphaFold DB; Jumper *et al.*, 2021 ▸) was announced by DeepMind and the EMBL–EBI (the European Bioinformatics Institute, part of the European Molecular Biology Laboratory; https://alphafold.ebi.ac.uk/). This database spans 20 species and includes, for example, the predicted 3D structure of *E. coli* HMBS (Jumper *et al.*, 2021 ▸; Tunyasuvunakool *et al.*, 2021 ▸). This includes a predicted structure for loop 45–57, which has often been missing in the experimental structures.

## 3. Discussion   

There are two principal themes in structural studies of this enzyme. The first is to unravel the details of the structural rearrangements during catalysis involving tetrapyrrole polymerization and release of a product (see Fig. 1 ▸) of precisely four units. The second theme is to relate the key amino-acid changes in the HMBS structure to the medical pathology acute intermittent porphyria (AIP). These two themes interrelate as the latter can be harnessed to inform the former, *i.e.* so as to trap intermediate states. Song *et al.* (2009 ▸) mapped a number of mutations that had been documented in the porphobilino­gen deaminase (PBGD) gene of patients suffering from AIP onto their crystal structure of human PBGD (PDB entry 3ecr).

The method of mutagenesis to fully trap an enzyme intermediate state such as ES_2_ has the danger that the enzyme is effectively blocked and cannot move towards extending to the next step and on towards release of the product. The danger then is that the crystal structures are artefactual or, if not exactly that, then at least not natural. A mutant such as K59Q (Niemann *et al.*, 1994 ▸) does not fully stop the enzyme at this second stage; rather, it slows the process down, which I think is a better approach to the natural state of the enzyme. In this case the X-ray diffraction experiment has to be sufficiently quick in its measurements to capture this accumulating population (Helliwell *et al.*, 1998 ▸).

An alternative to time-resolved diffraction is to freeze-trap at the key stage of a sufficient accumulated intermediate, using the time sequence established by the flow-cell method. Alternatively, since the pre-formed crystal itself could restrict movements due to crystal packing, the consistency of the intermediate trapped structures could be explored in experiments repeated on different crystal forms.

Pluta *et al.* (2018 ▸) elegantly combine techniques to obtain results in solution using NMR for structural details and solution scattering for overall HMBS shape monitoring. These results ‘suggest a reaction mechanism with localized segmental dynamics, ruling out the domain-reorienting mechanism’. Their crystallization was directly of the ES_2_ enzyme substrate intermediate eluted from a chromatography column (see Fig. 1 of Pluta *et al.*, 2018 ▸). They explain that

These results (crystal structures PDB 5m7f and 5m6r based on 3ecr of Song *et al.*, 2009 ▸), combined with the SAXS and NMR experiments, allow us to propose a mechanism for the reaction progression that requires less structural rearrangements than previously suggested [*by Louie *et al.* (1992 ▸)*]: the enzyme slides a flexible loop (R255-V263) over the growing-product active site cavity…The large number of AIP-causing mutations occurring in this loop confirms the key relevance of this structural element not only for the reaction but also in disease.

For comparison purposes with *E. coli*, the sequence of this key loop Arg255–Val263 in the human enzyme would be Asn235–Gln243, encompassing Cys242, which is covalently attached to the cofactor ring C1 (see PDB entries 1ah5, 1ypn and 2ypn). It is this covalently linked cysteine (Cys261) that pulls the cofactor past the catalytic Asp99 (Asp84 in *E. coli*). Supplementary Figs. S4 and S5 of Pluta *et al.* (2018 ▸) show the crystals of E_holo_ and ES_2_, which are colourless.

## 4. Consistencies and inconsistencies in the crystal structures   

In addressing the goal of elucidating the mechanism of the enzyme, we can ask: what are the consistencies and inconsistencies in the crystal structures thus far? Here we are mainly addressing the group of crystal structures with PDB codes 1ypn, 5m6r, 7aaj, 7aak, 7cdo and 7ccz.

### 4.1. Consistency of the active-site structures of the ES_2_ intermediate   

**Figure 3:**
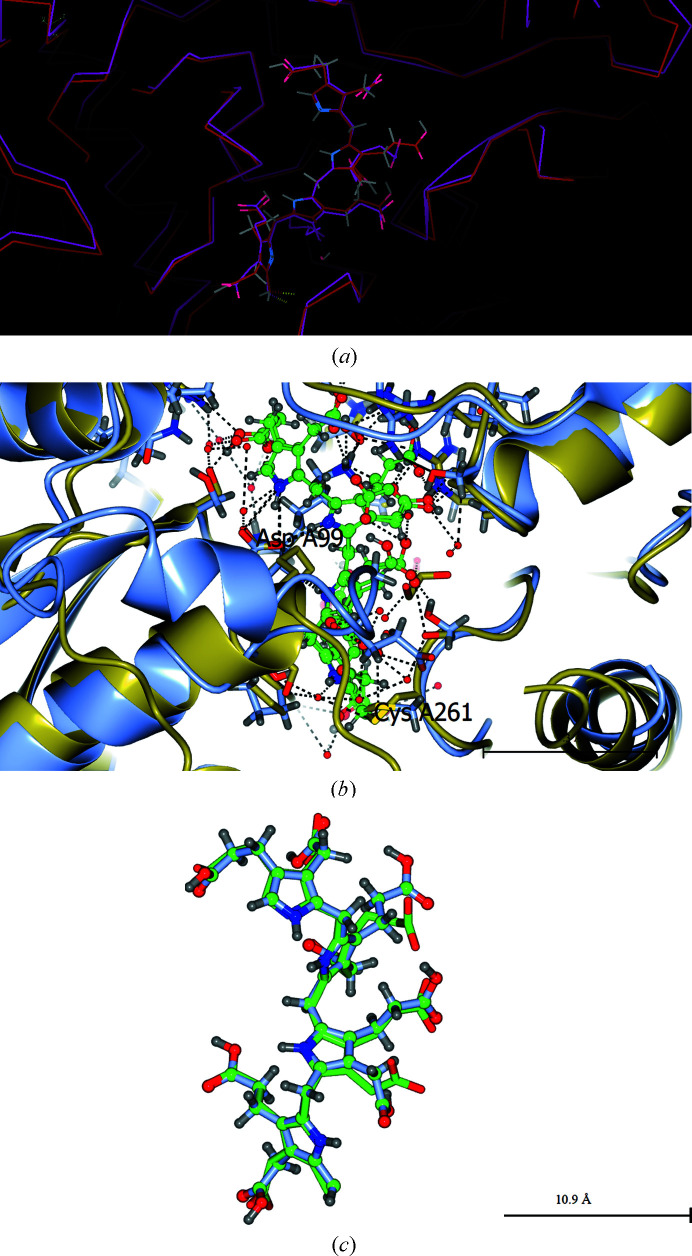
Best least-squares-calculated overlay of the cofactor plus ES_2_ for human HMBS, PDB entries 5m6r and human 7aak, in molecule *A*. Molecule *B* also shows a similarly good agreement between PDB entries 5m6r and 7aak. (*a*) was made with *Coot* (Emsley *et al.*, 2010 ▸), and (*b*) and (*c*) were made with *CCP*4*mg* (McNicholas *et al.*, 2011 ▸). All three show very similar orientations and show complementary information. Cys261 is covalently linked to the first ring of the cofactor. The least-squares-calculated overlay of PDB entries 5m6r and 7aak used the five amino acids centred on Asp99.

Firstly, Fig. 3 ▸ shows the best least-squares-calculated overlay of molecule *A* of the cofactor plus ES_2_ in PDB entries 5m6r and 7aak. These structures, which are both of human HMBS, are remarkably consistent considering that they were obtained by two different groups in two different space groups and one is an Arg173Trp mutant form of the enzyme. This mutation was chosen as it prevents the formation of ES_3_ [see Fig. 2(*d*) of Bustad *et al.* (2021 ▸), which is a mass spectrum showing only ES_2_]. These two crystal structures agree that the new pyrrole rings S1 and S2 occupy the original positions of the C1 and C2 rings of the DPM cofactor in the HMBS enzyme structure.

**Figure 4:**
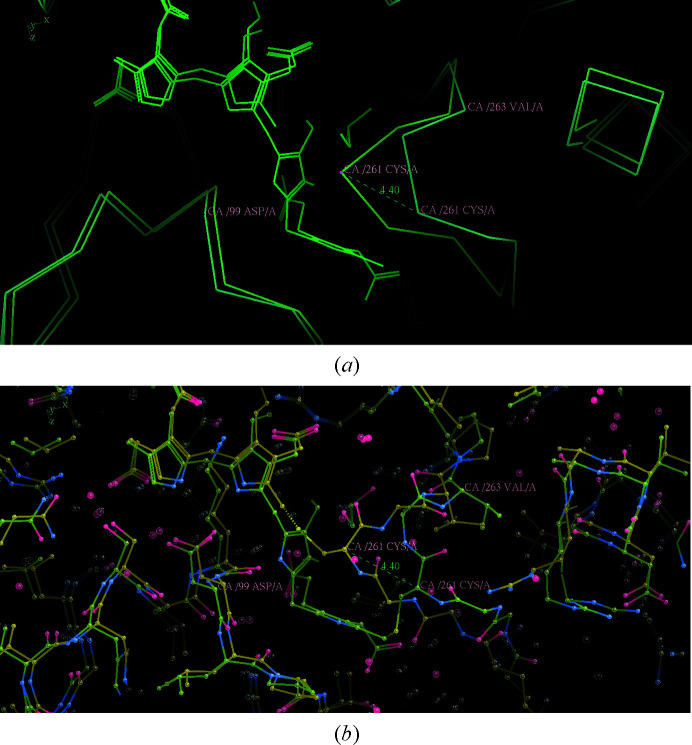
The motion of (human) Cys261 itself is largely responsible for pulling the cofactor to make room for the addition of two PBG molecules to form ES_2_ (Bustad *et al.*, 2021 ▸). The movement of Cys261 (PDB entries 7aaj and 7aak, molecules *A*) is 4.4 Å and that of Val263 is 0.8 Å. The movement of Asp99 is 0.5 Å. (*a*) shows the alpha carbons and the cofactors and ES_2_; (*b*) shows an identical view with all atoms.

An important observation of Bustad *et al.* (2021 ▸) is in the caption to their Supplementary Fig. S5, where they state that ‘Upon the movement of cofactor-binding loop from E_holo_ to ES_2_ only small change in Val263 can be detected’. This is shown in Fig. 4 ▸, which emphasizes that it is the motion of Cys261 itself that is largely responsible for pulling the cofactor to make room for the addition of two PBG molecules to form ES_2_.

**Figure 5:**
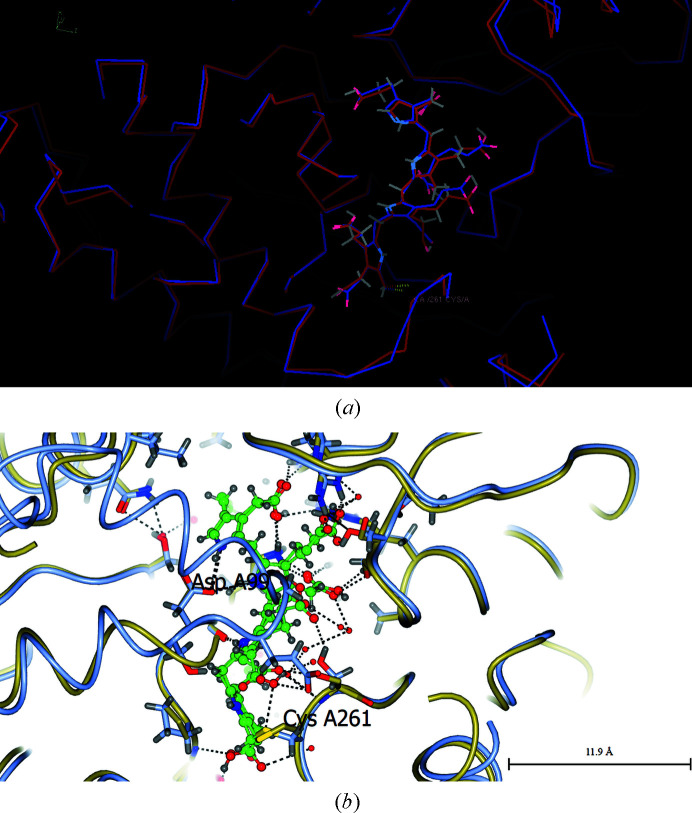
Best least-squares-calculated overlay of the cofactor plus ES_2_ for human HMBS, PDB entries 5m6r and human 7cd0, in molecule *A*. Molecule *B* shows very similar agreement. Cys261 is at the lower middle and thereby also identifies the first ring of the cofactor. Note that the iodinated PBG inhibitor is in molecule *B*. (*a*) was made with *Coot* (Emsley *et al.*, 2010 ▸) and (*b*) was made with *CCP*4*mg* (McNicholas *et al.*, 2011 ▸). Both show very similar orientations and show complementary information. Cys261 is covalently linked to the first ring of the cofactor. The least-squares-calculated overlay of PDB entries 5m6r and 7cd0 used the five amino acids centred on Asp99.

Fig. 5 ▸ documents similar agreement for the cofactor plus ES_2_ for PDB entries 5m6r and 7cd0. Similar agreement for ES_2_ is shown by PDB entry 7ccz.

### 4.2. Time-resolved diffraction experiment on the Lys59Gln mutant of HMBS to accumulate ES_2_   

**Figure 6:**
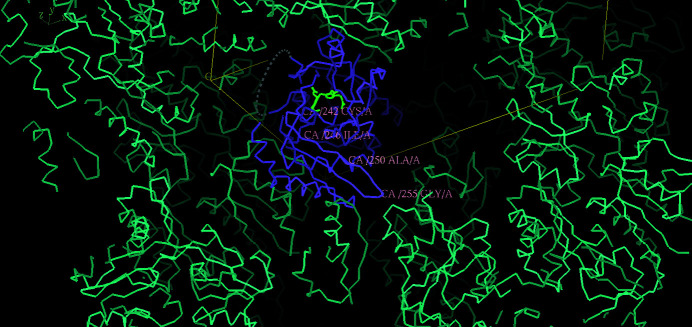
Crystal-packing diagram for *E. coli* (PDB entry 1ypn) showing the solvent channel directly above the 242–255 polypeptide loop of HMBS. Note that the lattice neighbour of Gly255 is Gly33 and residue 32 is a proline, *i.e.* it is unlikely to interfere with loop movement.

PDB entry 1ypn from the time-resolved study showed the growth of an extended electron density from the region of Asp84 and cofactor ring C2. Like those used for PDB entry 1ah5, the crystals used for PDB entry 2ypn were colourless before the flow of PBG over the crystals, with the cofactor in the active state. In the case of PDB entry 1ypn, after 2 h of PBG flow the crystals became pink/red (see Section 4.2.4 of Helliwell *et al.*, 1998 ▸). To achieve the full red colour at 12 h requires release of the product and cyclization of the tetrapyrrole. After the PBG supply had been turned off at the 2 h time point, a crystal structure was determined at 12 h. The extended electron density that grew in the active site by 2 h had disappeared, whilst the crystal at 12 h was red. How do we reconcile these observations with the agreed model (Figs. 3 ▸, 5 ▸ and 6 ▸) of the cysteine bond (Cys261 in human HMBS, corresponding to Cys242 in *E. coli* HMBS) to cofactor ring C1 pulling the growing pyrrole chain so that S1 and S2 occupy the sites originally occupied by C1 and C2? One option referred to in Helliwell *et al.* (1998 ▸) was that the red colour arises from the enzyme molecules on the crystal surface only and therefore the extended electron density that grew in the active site was only the arrival of PBG in the active site. Indeed, there was no bond to the C2 ring, which would support this; *i.e.* it was a pre-reactive species. The results shown in Figs. 3 ▸, 5 ▸ and 6 ▸ suggest a new possibility: that the reaction in the crystal from which PDB entry 1ypn was obtained had gone to EP, the cofactor had returned to its starting position, and the bond between S4 and the cofactor C2 ring would also have been broken. This is only viable if Cys242 and the nearby loop residues 243 onwards were able to move unrestrictedly, *i.e.* if there was a solvent channel directly above the loop. Otherwise, the loop would fight against the crystal packing and the crystal would most likely have broken up. Fig. 6 ▸ shows that there is a solvent channel directly above this loop. Against this second hypothesis is that we did not directly see sliding of the DPM cofactor or of the Cys242 associated with it (at most 0.1 Å at 8 min; see Fig. 4 of Helliwell *et al.*, 1998 ▸).

To return to the visual observations made in Section 4.2.4 of Helliwell *et al.* (1998 ▸): ‘In separate experiments involving the soaking of a wild-type HMBS crystal in a pot of solution the pink/red colour developed in similar fashion to the flow cell experiments.’ Furthermore,

After *ca.* 24 h the crystal showed distinct cracks and the surrounding solution also became gradually pink within 13–24 h, but markedly more slowly than the crystal (results of A. Hädener). These observations are consistent with formation of a cyclized oxidized product in the crystal, which may or may not be released from the active site. It cannot be ruled out, however, that non-enzymatic formation of tetrapyrrole in solution and subsequent cyclization and oxidation processes are followed by the absorption of the oxidized material by the crystal, thus concentrating it like a red dye.

It is also interesting that Azim *et al.* (2014 ▸) contains an extensive discussion of their cofactor states and colourations (pink as well as yellow). They noted an ‘intriguing pink colouration of the freshly purified protein which gradually changes to yellow over a 2–3-week period.’

### 4.3. What is the role of the loop 61–76 (human HMBS numbering)?   

PDB entries 4htg, 5m6r and 7aak are exceptions in that they show the ordering of this loop. Actually, however, PDB entries 5m6r and 7aak, which each have two HMBS molecules in their asymmetric units, have disordered loops in one of these two HMBS molecules. Yet both PDB entries 5m6r and 7aak have their two HMBS molecules with fully formed C1+C2+S1+S2 in their ES_2_ crystal structures. This suggests that the role of this loop is incidental to the catalytic sequence of events, at least up to ES_2_. However, Sato *et al.* (2021 ▸) report that ‘flexibility of this loop in the proximity of the active site appears to be involved in the binding of 2-I-PBG and the substrate, although no direct interactions between the loop (residues 58–69) and 2-I-PBG were observed’. This last sentence seems to be a little self-contradictory.

### 4.4. What can be said about the ES_2_ to ES_3_ step?   

As the experimental design in Bustad *et al.* (2021 ▸) involved the R173W mutant and they showed conclusively that ES_3_ was not produced, then it is compelling to agree with Bustad *et al.* (2021 ▸) that ‘the substrate elongation from ES_2_ to ES_3_ is crucially dependent on Arg173’. Secondly, since Pluta *et al.* (2018 ▸) actually crystallized ES_2_ for the wild-type enzyme and Fig. 3 ▸ shows the agreement between the two approaches, this lends further support to the crucial role of Arg173.

Sato *et al.* (2021 ▸) came the closest to a crystal structure of ES_3_; they reported that they ‘attempted crystallization and structure analysis of ES_3_ intermediate of HMBS, and successfully obtained its crystals. However, structural analysis of the ES_3_ intermediate has not yet been successful due to its instability.’ However, they make the very plausible conclusion from their several crystal structures that ‘Since 2-I-PBG is present at the same site in both structures (holo hHMBS and ES_2_), it is considered that each of the four substrate molecules binds to a single substrate-binding site in HMBS and is condensed consecutively on the DPM cofactor in four successive reactions.’ This assertion then provides a definite suggestion regarding ES_3_ and ES_4_ formation. Also, it should be considered that the step from ES_2_ to ES_3_ requires further rearrangement, which cannot happen in the crystal state. Therefore, ES_3_, stabilized somehow, may need to be formed before crystallization.

### 4.5. What can be said about how the product is released?   

**Figure 7:**
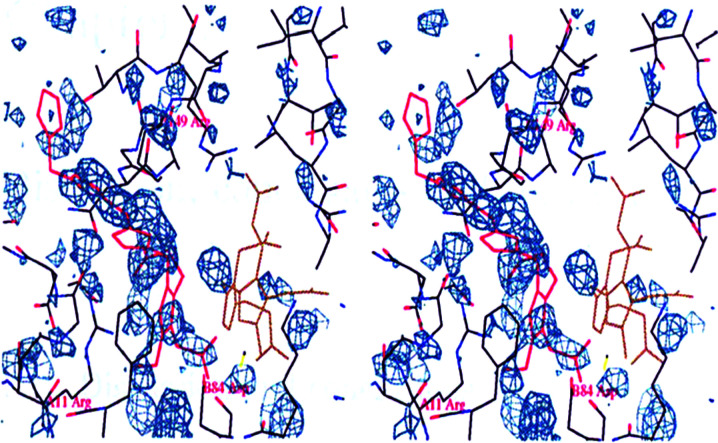
Stereoview of the modelling of the Michaelis complex EP (red) overlaid with the 2 h time point (*F*_o_ − *F*_c_) electron-density omit map contoured at 2.00σ. From Nieh (1997 ▸). The acetate and propionate side chains of the second, third and fourth pyrroles could not be placed due to insufficient detail in the density map.

Bung *et al.* (2014 ▸, 2018 ▸, 2019 ▸) undertook molecular-dynamics (MD) studies. Fig. 7 from Bung *et al.* (2018 ▸) and the associated text describe three possible routes for the formed product to exit the enzyme. Specifically, they state that ‘R167 acts as a gatekeeper for the HMB exit’. In his PhD thesis, Nieh offered a similar assertion based on the 2 h time-resolved experiment (Nieh, 1997 ▸):

The elongated peak passes the critical residues Arg149, which is important in forming ES_2_, and approaches Arg155, which is important in the ES_4_ to EP step, and then projects out into the solvent channel. Residues Arg149 and Arg155 in *E. coli* are equivalent to residues 167 and 173 in the human HMBS enzyme and mutation of these two residues can cause acute intermittent porphyria. The modelling of EP is shown in Fig. 6.13 [*reproduced here as Fig. 7 ▸*] with the final omit map overlaid. Note that only ring A gives a reasonably detailed fit.

### 4.6. Consistency given by combining different methods   

Bustad *et al.* (2021 ▸) elegantly show how high-resolution mass spectrometry allows direct analysis of the intermediates in order to directly plan the X-ray crystal structures. A weakness of an X-ray crystal structure is that where there is disorder, such as a mobile loop or a floppy tripyrrole or tetrapyrrole, there will be broken up or missing electron density. However, if the mass is directly measured then this datum assumes a special importance. This approach of measuring the mass was also performed by Sato *et al.* (2021 ▸).

The role of SAXS where there might be large-scale inter-domain movements, as originally proposed for HMBS by Louie *et al.* (1992 ▸), can be definitive and shows what is happening in solution. However, if the mechanism has been shown to be the localized movement of Cys261, without inter-domain movements, then SAXS will be insensitive to such localized changes.

The role of monitoring the colour, from colourless to pink to full red, is illustrated well in the efforts to unravel the mechanism of HMBS. The colour changes have been emphasized several times above.

X-ray crystal structures at these diffraction resolutions cannot show the H atoms of ionizable groups, or even at ultrahigh resolution if the side chains are a little mobile. [Non-ionizable groups can have their H atoms placed in calculated positions with confidence.] Since molecular-dynamics simulations propose a mechanism relying on the protonation of incoming PBG molecules by Arg26, and electrophilic addition and deprotonation in concert with Asp99 (Bung *et al.*, 2018 ▸, 2019 ▸), then experiments seeking to place these H atoms, and as a function of time, are the most challenging of all. Neutron crystallography of suitably trapped intermediate states through wet chemistry or freeze-trapping has not yet been applied. These experiments would require larger crystals to be grown than hitherto. It is also worth noting that the pH optimum of the *E. coli* HMBS enzyme is 7.4–8.0, yet the best crystallization conditions thus far were at pH 5.3. As Hädener *et al.* (1999 ▸) remark

The pH optimum for HMBS from *E. coli* is between 7.4 and 8.0, and the isoelectric point is 4.5. Within the optimal pH interval and for the overall reaction, the Michaelis constant is between 5 and 20 m*M*, and the turnover constant is of the order of 0.1 s^−1^ with respect to the formation of HMB.

Functionally relevant protonation states will require crystals that are grown under functionally appropriate conditions.

To summarize, the human HMBS crystallization conditions, and in one case the solution for SAXS measurements, were as follows.

Song *et al.* (2009 ▸) state in PDB entry 3ecr that the pH of their human HMBS crystallization was 8.0 (this was not stated in their paper).

The crystallization conditions used by Pluta *et al.* (2018 ▸) for human HMBS were between pH 6.5 and 7.2, and their SAXS solution was at pH 8.5.

The crystallization conditions used by Bustad *et al.* (2021 ▸) for human HMBS were at pH 5.1.

The crystallization conditions used by Sato *et al.* (2021 ▸) for human HMBS were at pH 8.3.

In addition to the extensive MD simulations of Bung *et al.* (2019 ▸) described above, MD calculations of the ES_2_ intermediate were also undertaken by Sato *et al.* (2021 ▸). These suggested that thermal fluctuation of the lid (residues 58–75, human numbering) and cofactor-binding loops causes the substrate recruitment and oligopyrrole chain shift needed for consecutive condensation. This suggestion relates to the observation by Bustad *et al.* (2021 ▸) that Cys261 is the locus of the movement of the cofactor during catalysis, but what is the ‘engine’ or energy to drive this movement? Perhaps Sato *et al.* (2021 ▸) have the answer, namely thermal fluctuation (of the lid and cofactor-binding loops). However, thermal fluctuations are random displacements rather than being purposeful in a particular direction. The MD simulations performed by Sato *et al.* (2021 ▸) required the protonation states of Glu223 and His160; at these diffraction resolutions these needed to be predicted, which was performed using the *H++* server (http://biophysics.cs.vt.edu). An evaluation of the predictive accuracy of several prediction servers has been undertaken by Fisher *et al.* (2009 ▸), who concluded that predicting histidine protonation was especially difficult. Sato *et al.* (2021 ▸) provide three movie files; the first two show the jittery nature of a wide suite of atomic motions and the third movie, of the ES_2_ in HMBS in skeletal format, very clearly shows the simulated dynamic role of loops.

## 5. Future directions   

Experimental capabilities for time-resolved diffraction have expanded considerably. The new extremely bright synchrotron-radiation sources such as those at MAX IV and ESRF will allow smaller protein crystal sizes. In the experiments of Helliwell *et al.* (1998 ▸) the *E. coli* HMBS crystals were typically 0.5 × 0.5 × 0.05 mm in size. The human HMBS crystals used by Sato *et al.* (2021 ▸) were ∼0.2 × 0.2 × 0.02 mm for the inhibitor-free holo form and ∼0.1 × 0.1 × 0.01 mm for the inhibitor-free ES_2_ intermediate. The diffusion time of PBG into the crystal for smaller crystals would be speeded up accordingly. These would also likely lead to better quality (less mosaic) freeze-trapping of crystals in intermediate states. This said, there is a growing awareness of examples of cryo-artefacts which must be guarded against, such as incorrectly placed side chains and their bound waters (see, for example, Halle, 2004 ▸).

The availability of high-quality protein fold and structure predictions across multiple species from AlphaFold DB (Jumper *et al.*, 2021 ▸; Tunyasuvunakool *et al.*, 2021 ▸) will allow further insights into the mobile loops in HMBS that have challenged experimental methods. Secondly, these predicted structures may well allow new crystallization constructs that can be used to explore different crystal packings and further crystallizations at the functional pH of the enzyme. Such crystallization constructs would allow new avenues to be explored to grow larger crystals for neutron crystallography, as well as the application of macroseeding methods (Chayen *et al.*, 2010 ▸).

The community of researchers interested in this fascinating enzyme is growing, as shown by the pace of entries in Table 1 ▸. I hope that this topical review proves useful to further expand this community.

## Supplementary Material

Supplementary Figures. DOI: 10.1107/S2053230X2100964X/va5043sup1.pdf

## Figures and Tables

**Table 1:** Summary of the structural data and core details for the HMBS crystal structures in the PDB as of mid-2021 (also included in this table is the entry in AlphaFoldDB; Jumper *et al.*, 2021 ▸)

PDB code (publication reference)	Biological source	Crystal parameters†	Title of PDB entry	Colour of crystal	The highest difference Fourier (*F*_o_ − *F*_c_) electron-density peak (viewed in *Coot*) and any specific comments therefrom‡	PDB Validation Report assessment (clashscore; my own specific comments of interest based on the PDB report)‡
1pda (Louie *et al.*, 1992 ▸, 1996 ▸)	*Escherichia coli*	1.76 Å; *P*2_1_2_1_2; 88, 75.9, 50.5 Å; *Z* = 1	*Structure of porphobilinogen deaminase reveals a flexible multidomain polymerase with a single catalytic site*	Yellow§, *i.e.* inactive form of the enzyme	10.6σ. The functionally interesting peak is peak 6 at −5.5σ on the second cofactor ring. It is stated that this ring is at 90% occupancy. The cofactor is in the inactive oxidized state. There are ten peaks above ±5.0σ, the *Coot* default threshold.	Clashscore 6. Significant negative electron-density difference density (3σ) on the DPM314A second ring is shown in the report.
1ah5 (Hädener *et al.*, 1999 ▸)	*Escherichia coli*	2.40 Å; *P*2_1_2_1_2; 88.47, 76.15, 50.79 Å; *Z* = 1	*Reduced form selenomethionine-labelled hydroxymethylbilane synthase determined by MAD*	Colourless, *i.e.* active form of the enzyme	7.0σ. There are seven peaks above ±5.0σ. The 7.0σ peak is close to the second ring of the cofactor, with no obvious explanation. Peaks 2 and 5 are in the mobile loop region, *i.e.* Leu61 and Pro40, respectively.	Clashscore 8
1ypn (Helliwell *et al.*, 1998 ▸)	*Escherichia coli*	2.40 Å; *P*2_1_2_1_2; 88.47, 76.15, 50.79 Å¶; *Z* = 1	*Reduced form hydroxymethylbilane synthase (K59Q mutant) crystal structure after 2 h in a flow cell determined by time-resolved Laue diffraction*	Turned red during the time-resolved diffraction sequence	No *F*_o_ − *F*_c_ peaks above 5.0σ.	Clashscore 8
2ypn (Nieh *et al.*, 1999 ▸)	*Escherichia coli*	2.3 Å; *P*2_1_2_1_2; 88.06, 75.73, 50.35 Å; *Z* = 1	*Hydroxymethylbilane synthase*	Colourless, *i.e.* the active form of the enzyme	5.4σ. This is the only peak and is located above the bridge between the C1 and C2 cofactor rings.	Clashscore 4
1gtk (Helliwell *et al.*, 2003 ▸)	*Escherichia coli*	1.66 Å; *P*2_1_2_1_2; 87.5, 75.9, 50.1 Å; *Z* = 1	*Time-resolved and static ensemble structural chemistry of hydroxymethylbilane synthase*	Colourless, *i.e.* the active form of the enzyme	11.1σ. There are 21 peaks above 5.0σ. These are unmodelled split-occupancy peaks, bound waters and some signs of radiation damage.	Clashscore 11. Significant negative electron-density difference density (3σ) on the DPM315A second ring and positive on the bridge between the two rings.
3ecr (Song *et al.*, 2009 ▸)	Human	2.18 Å; *P*2_1_2_1_2_1_; 71.61, 81.061, 109.211 Å; *Z* = 2	*Structure of human porphobilinogen deaminase*		−9.6σ at Glu76B (radiation damage?). There are 14 peaks above ±5.0σ. These are misoriented side chains (*e.g.* Trp201A) and unmodelled waters.	Clashscore 13
3eq1 (Gill *et al.*, 2009 ▸)	Human	2.8 Å; *P*2_1_2_1_2; 81.083, 104.435, 109.732 Å††; *Z* = 2	*The crystal structure of human porphobilinogen deaminase at 2.8 A resolution*		7.1σ. 13 peaks above ±5.0σ. These are misoriented side chains (*e.g.* Gln29B, Trp198B) and unmodelled waters. There is a 5.2σ peak at the side chain of Arg195B and O3 and O4 of DPM401B. There is another at 5.1σ near to Thr145B and DPM401B and likewise at 5.1σ for DPM400A.	Clashscore 10
4htg (Roberts *et al.*, 2013 ▸)	*Arabidopsis thaliana*	1.45 Å; *C*2; 141.573, 37.271, 55.069 Å††; *Z* = 1	*Porphobilinogen deaminase from Arabidopsis thaliana*		6.1σ. There are six peaks above 5.0σ. These are mismodelled side chains or unplaced waters, but two amino acids, Asp78A (−5.9σ) and Glu32A (−5.5σ), show radiation damage.	Clashscore 6
4mlq (Azim *et al.*, 2014 ▸)	*Bacillus megaterium*	1.6 Å; *P*2_1_2_1_2_1_; 53.01, 65.12, 96.78 Å; *Z* = 1	*Crystal structure of Bacillus megaterium porphobilinogen deaminase*		7.4σ. There are just four peaks, but the top three are at DPM401A. See Supplementary Fig. S1. The interpretation of what these mean is unclear. Azim and coworkers model two conformations of the cofactor at 50% and their paper carries an extensive discussion of the cofactor states and colourations (pink as well as yellow and colourless).	Clashscore 2
4mlv (Azim *et al.*, 2014 ▸)	*Bacillus megaterium*	1.46 Å; *P*2_1_2_1_2_1_; 53.32, 65.78, 97.21 Å; *Z* = 1	*Crystal structure of Bacillus megaterium porphobilinogen deaminase*		9.0σ. There are 23 peaks above 5σ. These are mainly unmodelled split-occupancy side chains and possible bound waters. Peak 8 next to Val61A is an extended feature (blob); peaks 18 and 23 are similar.	Clashscore 3. The PDB Validation Report ligands and difference density show quite a number of peaks over both DPMs.
5ov5 (Guo *et al.*, 2017 ▸)	*Bacillus megaterium*	1.81 Å; *P*2_1_2_1_2_1_; 49.16, 62.7, 91.83 Å; *Z* = 1	*Bacillus megaterium porphobilinogen deaminase D82E mutant*		8.2σ. There are 13 peaks above 5.0σ. These are mainly unmodelled bound waters. There is evidence of radiation damage at Glu307A, Glu290A, Met175A and Cys241A.	Clashscore 2
5ov6 (Guo *et al.*, 2017 ▸)	*Bacillus megaterium*	1.81 Å; *P*2_1_2_1_2_1_; 48.99, 62.69, 91.26 Å; *Z* = 1	*Bacillus megaterium porphobilinogen deaminase D82N mutant*		9.2σ. Four peaks. All four are unmodelled bound waters.	Clashscore 2
5ov4 (Guo *et al.*, 2017 ▸)	*Bacillus megaterium*	2.8 Å; *P*2_1_2_1_2_1_; 49.092, 62.487, 91.391 Å††; *Z* = 1	*Bacillus megaterium porphobilinogen deaminase D82A mutant*		5.1σ. Just two peaks, which look like side-chain disorders.	Clashscore 3
5h6o (T. Funamizu, M. Chen, Y. Tanaka, K. Ishimori & T. Uchida, unpublished work)	*Vibrio cholerae*	2.7 Å; *I*4_1_22; 94.23, 165.34 Å; *Z* = 1	*Porphobilinogen deaminase from Vibrio cholerae*		6.3σ. Four peaks; three are perhaps bound waters, but being rather close to the protein may be series-termination errors. One is an unmodelled ‘blob’.	Clashscore 7
5m6r (Pluta *et al.*, 2018 ▸)	Human	2.7 Å; *P*2_1_; 68.91, 81.2, 79.69 Å, 93.01°; *Z* = 2	*Human porphobilinogen deaminase in complex with reaction intermediate*	Colourless crystal. See Supplementary Fig. S5 of Pluta *et al.* (2018 ▸).	6.7σ. 15 peaks. These are unmodelled bound waters or indistinct blobs. They may instead be series-termination errors.	Clashscore 1. The two tetrapyrroles are high-quality (2*F*_o_ − *F*_c_) fits.
5m7f (Pluta *et al.*, 2018 ▸)	Human	2.78 Å; *P*2_1_; 70.42, 80.95, 76.22 Å, 95.7; *Z* = 2	*Human porphobilinogen deaminase in complex with DPM cofactor*	Colourless crystal. See Supplementary Fig. S4 of Pluta *et al.* (2018 ▸).	7.0σ. Seven peaks. The top peak is a fairly extensive blob (Supplementary Fig. S2), as are peaks 3, 4 and 7. The latter two are possibly glimpses of the mobile loop 58A–74A.	Clashscore 2
7aaj (Bustad *et al.*, 2021 ▸)	Human	1.8 Å; *P*2_1_2_1_2_1_; 81.163, 84.608, 108.913 Å††; Z = 2	*Human porphobilinogen deaminase in complex with cofactor*		6.4σ. 17 peaks. The top two peaks form a fairly extended blob, as do peaks 5 and 6. Perhaps glycerol? The remainder are side chains that could be modelled slightly better or possible bound waters.	Clashscore 4. The PDB Validation Report mentions that ‘The analyses of the Patterson function reveals a significant off-origin peak that is 23.61% of the origin peak, indicating pseudo-trans­lational symmetry (tNCS)’.
7aak (Bustad *et al.*, 2021 ▸)‡‡	Human	1.7 Å; *P*2_1_2_1_2_1_; 81.241, 86.127, 107.39 Å††; *Z* = 2	*Human porphobilinogen deaminase R173W mutant crystallized in the ES_2_ intermediate state*		6.5σ. 18 peaks. These are side chains that could be modelled slightly better, or possibly bound waters or split-occupancy glycerol. There is evidence of radiation damage at Cys247A.	Clashscore 3. As with 7aaj, mention of tNCS. The two tetrapyrroles are high-quality (2*F*_o_ − *F*_c_) fits.
7cd0 (Sato *et al.*, 2021 ▸)‡‡	Human	2.31 Å; *P*2_1_2_1_2_1_; 81.416, 81.366, 108.85 Å††; *Z* = 2	*Crystal structure of the 2-iodoporphobilinogen-bound ES_2_ intermediate form of human hydroxymethylbilane synthase*		7.1σ. Six peaks above 5.0σ. One peak is a side chain that could be remodelled. The others are perhaps series-termination peaks; anyway, it is not obvious how to model them.	Clashscore 7. Also mention of tNCS. The two tetrapyrroles are high-quality (2*F*_o_ − *F*_c_) fits. The 2-iodoporphobilino­gen (2*F*_o_ − *F*_c_) is a bit broken up on the two, non-iodo, side chains (even though contoured at 0.7 r.m.s.d.) but is OK.
7ccy (Sato *et al.*, 2021 ▸)§§	Human	2.40 Å; *P*2_1_2_1_2_1_; 74.011, 81.237, 109.097 Å††; *Z* = 2	*Crystal structure of the 2-iodoporphobilinogen-bound holo form of human hydroxymethylbilane synthase*		There are no peaks above 5.0σ.	Clashscore 7. Also mention of tNCS. The 2-iodoporphobilinogen (2*F*_o_ − *F*_c_) here is completely well formed (compared with 7cd0).
7ccz (Sato *et al.*, 2021 ▸)	Human	1.79 Å; *P*2_1_2_1_2_1_; 81.454, 79.542, 108.678 Å††; *Z* = 2	*Crystal structure of the ES_2_ intermediate form of human hydroxymethylbilane synthase*		5.9σ. Nine peaks above 5.0σ. Arg22A could be remodelled (peak 4). The other peaks may be bound waters, but most are too close to the protein atoms and if placed would lead to clashes.	Clashscore 4. No tNCS indicated, although the space group and unit cell are very similar to those of 7cd0. The two tetrapyrroles are high-quality (2*F*_o_ − *F*_c_) fits.
7ccx (Sato *et al.*, 2021 ▸)	Human	1.84 Å; *P*2_1_2_1_2_1_; 70.411, 80.815, 109.194 Å ††; *Z* = 2	*Crystal structure of the holo form of human hydroxymethylbilane synthase*		6.55σ. There are six peaks above 5.0σ. These are split-occupancy or unmodelled waters in nonfunctional locations.	Clashscore 4
						
AlphaFoldDB (jointly with the EMBL–EBI) (Jumper *et al.*, 2021 ▸; Tunyasuvunakool *et al.*, 2021 ▸)	*Escherichia coli*		*E. coli hydroxymethylbilane synthase*			

† Resolution; space group; unit-cell parameters; *Z*.

‡ The PDB Validation Report concerns the derived model and not the details that have not been modelled. The *F*_o_ − *F*_c_ map is easily inspected in the molecular-graphics visualization system *Coot* (Emsley *et al.*, 2010 ▸), which was used to describe the unmodelled peaks in this table.

§ A nice picture of a yellow HMBS crystal is shown in Supplementary Fig. 3 of Azim *et al.* (2013 ▸).

¶ These were set to the unit-cell parameters of PDB entry 1ah5. At 12 h the unit-cell parameters were 87.52, 75.92, 50.12 Å. This was a monochromatic data set to 2.0 Å resolution measured on BM14 at ESRF.

†† These are likely not at a precision of three decimal places.

‡‡ I made calculational checks of PDB entries 7aak and 7cd0, where tNCS was indicated in the PDB Validation Report. I checked their space groups with *Zanuda* (Lebedev & Isupov, 2014 ▸) and confirmed their correctness.

§§ Raw diffraction-image data for PDB entry 7ccy were held at in the XRDa at Protein Data Bank Japan, which meant that I was able to process with them with *iMosflm* to a slightly better resolution of 2.2 Å instead of 2.4 Å. I confirmed that the electron-density maps were the same. There were also 23 Gbytes of raw diffraction-image data for PDB entry 7cd0. At the present time, due to the block structure of the files (32 × 800 Mbytes), I was unable to process these diffraction images myself.
