# Supplementary material for: The crystal structures of the enzyme hydroxymethylbilane synthase, also known as porphobilinogen deaminase
Source: Acta Crystallogr F Struct Biol Commun. 2021 Oct 19;77(Pt 11):388–98. doi: 10.1107/S2053230X2100964X (PMC8561815; doi:10.1107/S2053230X2100964X)
Supplement: Supplementary file 1 [file f-77-00388-sup1.pdf]

Acta Cryst F
STRUCTURAL BIOLOGY COMMUNICATIONS

Volume 77 (2021)

Supporting information for article:

The crystal structures of the enzyme hydroxymethylbilane synthase, also known as porphobilinogen deaminase

John R. Helliwell

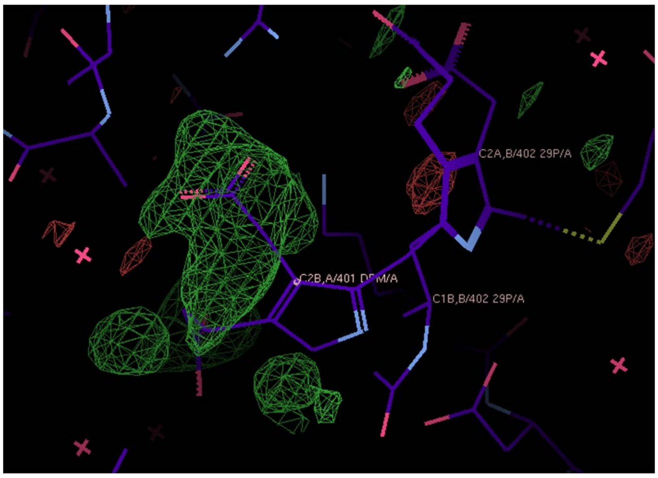

Figure S1 (Fo-Fc) difference electron density at DPM401A of PDB 4mlq contoured at 3σ. Azim et al 2014 explain though that “the structure of the enzyme in which the cofactor has undergone more extensive oxidation is presented in 4mlq compared with 4mlv”.

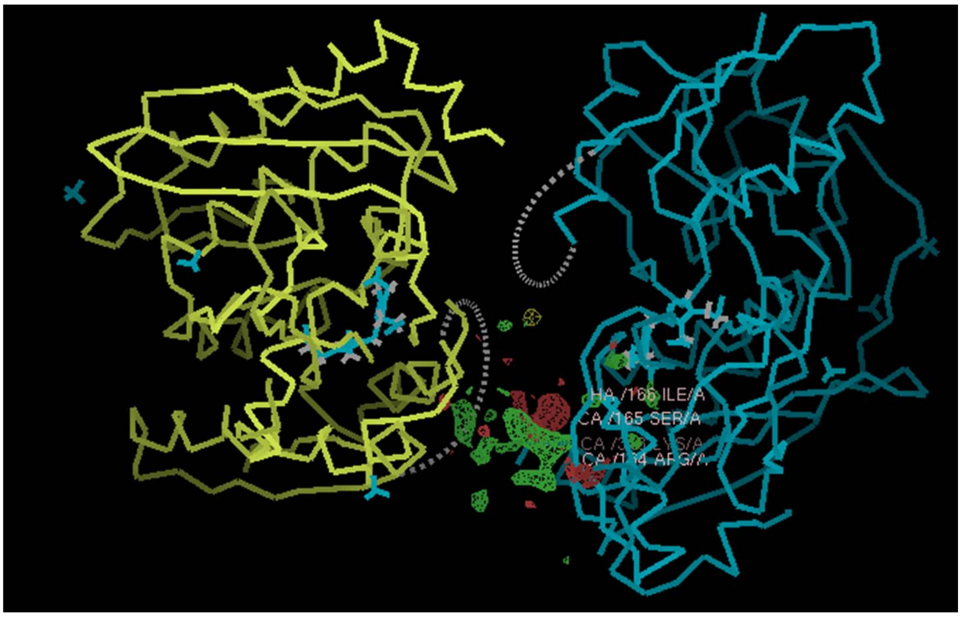

Figure S2 (Fo-Fc) difference electron density top peak (7.0σ) for PDB 5m6r contoured at 3σ.
